# Noma finally recognised as a neglected tropical disease

**DOI:** 10.1371/journal.pntd.0012177

**Published:** 2024-05-30

**Authors:** Stuart Ainsworth

**Affiliations:** 1 Department of Infection Biology and Microbiomes, Institute of Infection, Veterinary and Ecological Sciences, University of Liverpool, Liverpool, United Kingdom; 2 International Noma Network (www.internationalnomanetwork.org); George Washington University Medical Center, UNITED STATES

## Abstract

In December 2023, after decades of tireless advocacy from stakeholders and partners, the World Health Organization (WHO) gave noma the long overdue recognition as a neglected tropical disease. The significance of this official recognition cannot be overstated, and it is hoped this will serve as a turning point in our battle against this devastating disease.

Noma, an oral-facial gangrene, primarily affects chronically malnourished children aged between 2 and 6 years who live in conditions of extreme poverty [1]. Figures for the global incidence suggest 140,000 people a year may suffer from noma [2]. However, these figures are approaching 30 years old, with evident limitations noted [3]. The disease is currently primarily reported in Africa and Asia, with sporadic cases reported in other regions [3]. Noma can progress rapidly over a few days, and without prompt intervention, the fatality rate is thought to be as high as 90% [4]. The disease destroys facial tissues, often including the lips, cheeks, and nose, leaving survivors with significant facial deformity and scarring [5]. This often results in impaired eating, breathing, and speech. Due to their visible facial sequelae, survivors are frequently socially stigmatized and discriminated against, often missing out on key social and developmental opportunities, most commonly education [6]. The impacts of noma on populations, when estimated using disability-adjusted life years (DALYs) are substantial (1.7 to 7.8 million DALYs), comparable and in many cases surpassing other recognised neglected tropical diseases [7].

Many tropical diseases claim the title of “the most neglected of neglected tropical diseases,” but there is little doubt that noma fits this title better than any other. To illustrate just how little we know about noma, a recent scoping review of research publications on noma from 1843 to 2021, nearly 2 centuries, returned a total of 147 studies, less than 1 per year [8]. The vast majority of studies identified consisted of case reports or surgical approaches for improving sequelae. While risk factors for noma, such as chronic malnourishment, poor oral hygiene, and poor access to health and hygiene services, are well established, our understanding of many of the most basic aspects of the disease remains unknown [9]. Although recent efforts have been made to determine the epidemiology of noma in select regions [10], we still do not know with any certainty the aetiological causes or immunological conditions required for its establishment and progression.

Over the last 2 decades, momentum to improve the international status and awareness of noma has been building. Charities such as Hilfsaktion Noma and Médecins Sans Frontières have taken up the mantle in actively tackling the disease in affected areas, as well as providing funding for advocacy and raising awareness of the plight of sufferers and survivors. Individuals such as Leila Srour and the late Klaas Marck have been instrumental in raising international awareness and recognition of noma through relentless personal campaigning spanning decades [11,12]. The Nigerian government has been and remains highly proactive in supporting attempts to reduce its noma burden, with 2 dedicated noma hospitals within the country, in Sokoto and, more recently, in Abuja.

Noma has suffered from a chronic lack of resources, research, and understanding, which has led to it being actively ignored or fading from consciousness by the international community. As noma is noncommunicable and affects the most vulnerable and voiceless in society, there has been little incentive to tackle the disease by governments or international health agencies. Some governments actively deny noma exists within their populations despite evidence to the contrary. Knowledge of noma by medical professionals and front-line healthcare workers is extremely poor, leading to frequent misdiagnosis of the early signs of the disease, when simple intervention can prevent mortality and reduce morbidity [13]. Even among tropical disease specialists, noma is frequently unknown, with advocates often encountering blank faces or “do you mean the restaurant?” when asked about noma. Indeed, as recently as 2020, *PLOS Neglected Tropical Diseases* overlooked noma in an editorial entitled “What constitutes a neglected tropical disease?” [14,15].

In this context, the importance of noma being added to the World Health Organization (WHO)’s list of neglected tropical diseases cannot be overstated. The listing finally provides the international stage noma so desperately and rightly deserves and crucially provides credibility for noma in the eyes of governments, international health agencies, and funders. This will enable access to substantial funding and resource opportunities that were previously out of reach for noma stakeholders.

However, the noma community must act on this opportunity; a WHO neglected tropical disease listing is not an automatic golden ticket to funding and resources. Instead, it is a tool to leverage action. Through inaction, diseases can (and have) been removed prematurely from the list, such as snakebite in 2013 (since returned in 2017), which proved disastrous for funding [16]. Noma stakeholders must continue to effectively cooperate to maximize the opportunity this listing enables in order to translate it into tangible and sustained improvements to communities most at risk. In particular, researchers with multidisciplinary expertise and cutting-edge resources currently missing within the noma community, particularly immunologists and microbiologists, must be actively recruited to stimulate research to fill major gaps in our basic understanding of noma. This will enable the accelerated generation and development of much-needed noma-tailored clinical and public health tools based on a solid understanding of disease biology.

Noma is entirely treatable and preventable. Its former presence in Europe in the 19th century and subsequent eradication, thought to be due to improved nutrition, healthcare, and living conditions, and its continued existence in the 21st century, stand testament to the shocking ongoing neglect of the world’s poorest communities [12]. While it is likely that noma will persist for the foreseeable future, we can say with near certainty that the listing of noma by WHO will mean we are likely to generate more knowledge and understanding of noma over the next decade than we have over the last 2 centuries, but only if we can collectively seize this opportunity.
